# Design of dual ligands using excessive pharmacophore query alignment

**DOI:** 10.1186/1758-2946-4-S1-O11

**Published:** 2012-05-01

**Authors:** Daniel Moser, Joanna Wisniewska, Steffen Hahn, Estel la Buscató, Franca-Maria Klingler, Janosch Achenbach, Bettina Hofmann, Dieter Steinhilber, Ewgenij Proschak

**Affiliations:** 1 Institute of Pharmaceutical Chemistry, LiFF/OSF/ZAFES, Goethe University, Frankfurt am Main, Germany, Max-von-Laue Str.9, D-60438, Frankfurt/M, Germany

## 

Dual- or multi-target ligands have gained increased attention in the past years due to several advantages, including more simple pharmacokinetic and phamarcodynamic properties compared to a combined application of several drugs. Furthermore multi-target ligands often possess improved efficacy [1]. We present a new approach for the discovery of dual-target ligands using aligned pharmacophore models combined with a shape-based scoring. Starting with two sets of known active compounds for each target, a number of different pharmacophore models is generated and subjected to pairwise graph-based alignment using the Kabsch-Algorithm [2,3]. Since a compound may be able to bind to different targets in different conformations, the algorithm aligns pairs of pharmacophore models sharing the same features which are not necessarily at the exactly same spatial distance. Using the aligned models, a pharmacophore search on a multi-conformation-database is performed to find compounds matching both models. The potentially “dual” ligands are scored by a shape-based comparison with the known active molecules using ShaEP [4].

Using this approach, we performed a prospective fragment-based virtual screening for dual 5-LO/sEH inhibitors. Both enzymes play an important role in the arachidonic acid cascade and are involved in inflammatory processes, pain, cardiovascular diseases and allergic reactions [5,6]. Beside several new selective inhibitors we were able to find a compound inhibiting both enzymes in low micromolar concentrations. The results indicate that the idea of aligned pharmacophore models can be successfully employed for the discovery of dual-target ligands.
